# Detection of Blood Clots Using a Whole Stent as an Active Implantable Biosensor

**DOI:** 10.1002/advs.202304748

**Published:** 2024-02-11

**Authors:** Mahmut Talha Kirimi, Daniel Hoare, Michael Holsgrove, Jakup Czyzewski, Nosrat Mirzai, John R. Mercer, Steve L. Neale

**Affiliations:** ^1^ Centre for Medical and Industrial Ultrasonics James Watt School of Engineering University of Glasgow Glasgow G12 8QQ UK; ^2^ Institute of Cardiovascular and Medical Sciences/British Heart Foundation University of Glasgow Glasgow G12 8QQ UK; ^3^ BioElectronics Unit College of Medical Veterinary and Life Sciences University of Glasgow Glasgow G12 8QQ UK

**Keywords:** 3D printing, blood clot, cardiovascular disease, impedance sensor, restenosis, smart Stent

## Abstract

Many cardiovascular problems stem from blockages that form within the vasculature and often treatment includes fitting a stent through percutaneous coronary intervention. This offers a minimally invasive therapy but re‐occlusion through restenosis or thrombosis formation often occurs post‐deployment. Research is ongoing into the creation of smart stents that can detect the occurrence of further problems. In this study, it is shown that selectively metalizing a non‐conductive stent can create a set of electrodes that are capable of detecting a build‐up of material around the stent. The associated increase in electrical impedance across the electrodes is measured, testing the stent with blood clot to mimic thrombosis. It is shown that the device is capable of sensing different amounts of occlusion. The stent can reproducibly sense the presence of clot showing a 16% +/−3% increase in impedance which is sufficient to reliably detect the clot when surrounded by explanted aorta (one sample *t*‐test, *p* = 0.009, *n* = 9). It is demonstrated that this approach can be extended beyond the 3D printed prototypes by showing that it can be applied to a commercially available stent and it is believed that it can be further utilized by other types of medical implants.

## Graphical abstract

In Stent Restenosis and In Stent Thrombosis are major issues that trouble patients after stent deployment. In order to deal with these issues, researchers have been improving stents over multiple stent generations. Lately, smart stents have been introduced to monitor stages of these diseases. In this paper, it is shown that the whole stent itself can be converted into a biosensor and can be used for monitoring and diagnostic purposes.

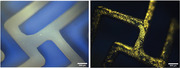

## 1 Introduction

### 1.1 Cardiovascular Disease and Treatment

The world health organization (WHO) reports a third of all deaths are a result of cardiovascular disease (CVD).^[^ ^1^ ^]^ This disease affects 17.9 million pA^−1^ and is largely driven by atherosclerosis; the build‐up of fibrofatty tissue over time within an artery.^[^ ^2^ ^]^ This accumulation initiates an inflammatory cascade that results in the growth of the lesion, which impinges blood flow through the blocked area^[^ ^3^ ^]^ and leads to downstream tissue ischemia. Overtime, a plaque forms that restricts blood flow and can rupture into the blood stream causing occlusive thrombosis, emboli formation that causes the majority of myocardial infarctions (heart attacks) or cerebral strokes.^[^ ^4^ ^]^

Prevention of CVD is at the forefront of global healthcare. Interventions such as cessation of smoking, a healthy diet and active lifestyle all decrease the likelihood of the disease.^[^ ^5^, ^6^ ^]^ Through these healthy changes the impact of hypertension, obesity and incident of type II diabetes can be reduced. The use of lipid lowering medications such as statins and anti‐hypertension drugs reduce cholesterol and blood pressure. These can be used to maintain vascular health, yet vessels may still block.

Percutaneous coronary intervention (PCI) is commonly employed to reopen the blocked artery(s) of the heart when the critical blockage limits the flow of blood within the coronary circulation.^[^ ^7^ ^]^ The coronary arteries are accessed using a series of catheters from a peripheral access location, usually the radial or femoral artery. Devices such as pressure / flow catheters, calculate the fractional flow reserve (FFR) or the degree of reduced flow across the impinging lesion while intravascular ultrasound (IVUS) images quantify the type of plaque.^[^ ^8^, ^9^, ^10^ ^]^ For lesions with an FFR below ≈0.7 (1.0 being normal) a catheter‐based balloon is threaded up to the point of the blockage, expanded, pushing back the atherosclerotic plaque. A metal scaffold called a stent is then used to permanently hold back the plaque and keep the vessel lumen patent.

Insertion of a stent to treat atherosclerosis leads to damage of the fine inner endothelial layer of the artery. This is caused by the hard strut of the stent pushing into the delicate arterial wall and denuding the inner layer of endothelial cells. This can induce acute in‐stent thrombosis (IST) as the circulating blood cells are exposed to the underlying medial vascular smooth muscle cells. Overtime this leads to a wound response and formation of a neointima. Excessive cellular growth will cause a new blockage termed – in stent restenosis (ISR).^[^ ^11^ ^]^ This response causes a complex cascade of cellular growth and inflammatory cytokines that drives vascular smooth muscle cell hyperplasia (VSMCs) and re‐narrowing that can present clinically as chronic angina.^[^ ^12^, ^13^, ^14^ ^]^ IST is particularly dangerous within the coronary circulation of the heart as they can lead to the myocardial infarctions. Some parts of the clot that break off (emboli) can also enter the cerebral circulation to cause strokes. The clot can form through aggregation of platelets in the stent both acutely <30 days post implantation or >12 months post implantation.^[^ ^15^ ^]^

There have been three major iterations of stenting technologies in order to overcome the issues that are introduced with stenting; 1) bare metal stents (BMS), 2) drug eluting stents (DES) and 3) bioabsorbable stents (BRS).^[^ ^16^ ^]^ The latest version that entered the market is Bioresorbable Vascular Scaffold (BVS).^[^ ^17^ ^]^ These stents were made from bioresorbable compounds (Poly‐L‐Lactic Acid ‐PLLA) that over time dissolve releasing their drug and then disappearing to allow for re‐endothelization.^[^ ^18^ ^]^ These devices struggled from lack of radial force within the stents as certain areas of the stent dissolved. Food and Drug Administration (FDA) approved the use of ABSORB GT1 BVS System in July 2016, a bioabsorbable stent, however later in 2017 a statement from the FDA raised concerns on the use of the BVS, which followed by the removal of the device from the world market in September 2017.^[^ ^19^ ^]^

The issues such as late ISR still persist even though there are advancements in stent technologies such as improvements in drug elution kinetics by improving the polymers, which effects the rate at which a drug is eluted from the stent.^[^ ^20^, ^21^, ^22^, ^23^ ^]^ A different approach can be beneficial when it comes to combat the issues brought by the stenting.

### 1.2 Impedance Detection

The use of electrical impedance for the detection of cells and their behavior was shown by the seminal work of Giaver and Keese.^[^ ^24^, ^25^, ^26^, ^27^ ^]^ The passing of an alternating current between the electrodes in the presence of cells causes the electric field to be deflected, and the strength of this interaction is dependent upon the frequency of the applied AC and the physical properties of the cell in the field. In the case of low frequency, the majority of the field passes paracellular while at high frequency the majority passes transcellular.^[^ ^28^ ^]^ Other researchers, along with our group, have shown that through the measurement of impedance, different vascular pathologies can be identified.^[^ ^29^ ^]^ Furthermore, the use of impedance‐based sensors has successfully detected blood clots.^[^ ^30^ ^]^ However, to date, this has been on sensors purely for in vitro research or integration into a stent. To this extent we propose the use of a whole 3D printed stent which has been selectively metallized for the detection of cellular material in a restenotic stent replicate.

### 1.3 Future Developments

Integration of a sensor into the stent has been hypothesized to include temperature, pressure and pH sensors.^[^ ^31^ ^]^ Many of these devices struggle from the integration of bulky electronics into the fine structure of the stent that may be just a few millimeters in diameters. To overcome this, researchers have investigated the use of the stent as a component in order to reduce the electronics size. The “stentenna” is an integrated pressure sensor with novel antenna; with the stent itself acting as the antenna.^[^ ^32^, ^33^ ^]^ Others have used the natural geometry of the stent as a method for the detection of pressure through the strain on the device.^[^ ^34^ ^]^

Our group have put forward the use of SMART stents as a novel self‐reporting device for discriminating cells type and addressing ISR using electrical impedimetric changes.^[^ ^35^, ^36^, ^37^, ^38^ ^]^

Here, we provide evidence of a way to use rapid prototyping to evaluate a smart implantable device. We show that these stent devices can be converted from being inert to a working biosensor by metallizing specifically targeted segments to create a set of electrodes that utilizes the changes in electrical impedance due to cellularity.

## 2 Results

### 2.1 COMSOL Simulations

**Figure 1:**
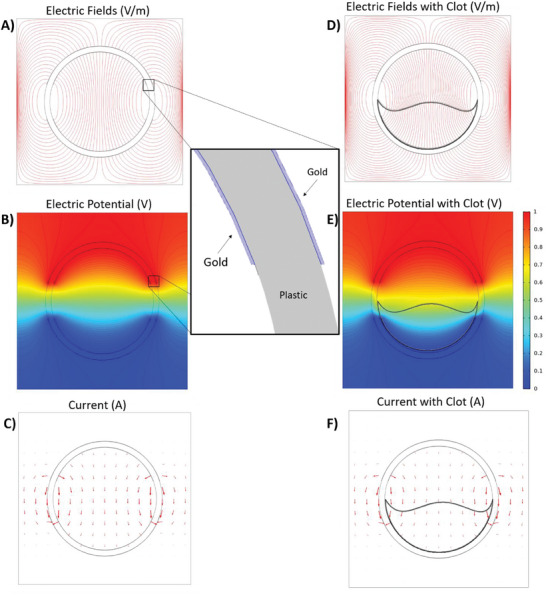
Cross section of a stent sensor with and without interference of a material in its mid‐section; A) and D) shows electric field lines with A) showing a clear stent and D) showing a stent with a clot, B) and E) electric potential, B) with a clear stents and E) with a clot, C) and F) current, E) with a clear stent and F) with a clot present. The highlighted area on A) and B) are uniform within all simulations, which shows the thin layer of gold on both sides of plastic non‐conductive base. Additionally, highlighted area on (D), (E) and (F) indicates the thrombi area. Electric Potential (B–E) graphs show the material in the device has an effect on the voltage distribution which in turn causes significant changes on the impedance which can be detected by the stent for diagnostic purposes.

The results of COSMOL Simulations are given in **Figure** **1**.

### 2.2 Investigating Blood Clot

**Figure 2:**
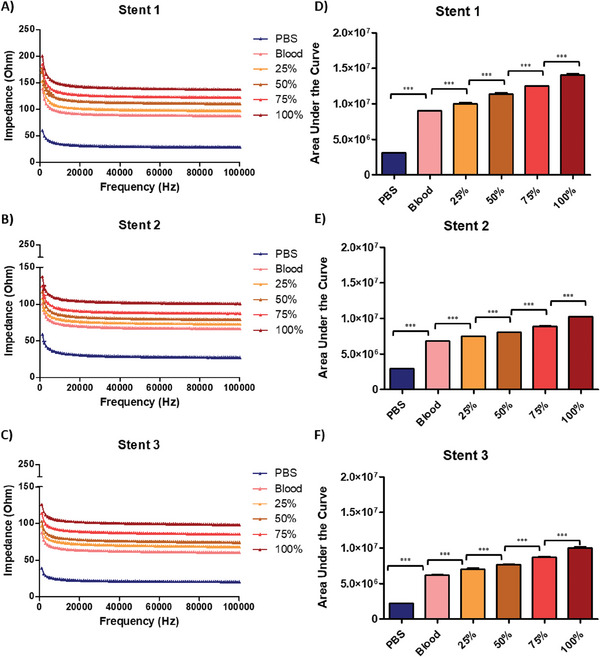
(A–C) showing impedance over a frequency spectrum that ranges from 1 kHz to 100 kHz of the stents while (D–F) shows the area under the curve results of three different stents that show significant differences between control and experimental groups within each stent`s group itself when compared to each other using one way ANOVA (*p* < 0.001). The area under the curve data that is calculated from the impedance measurements of the frequency sweep increased in correspondence with occlusion as an indicator to show the stent`s capability to detect the changes in the amount of occlusion (n = 3).

**Figure 3:**
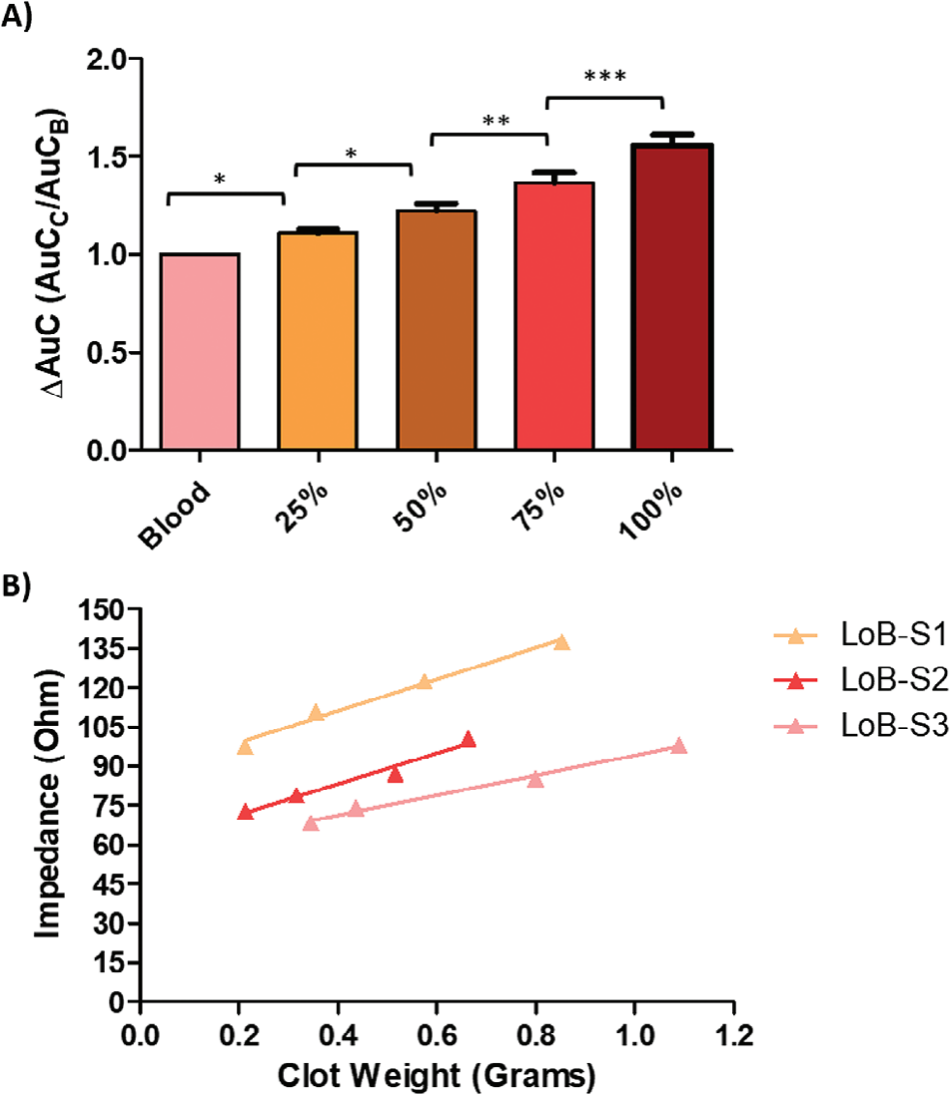
Summary of replicate whole stent data for blood clot detection A) Ratio change between each stent group where the control group (Blood) was set as 1 and the experimental groups were divided by the control group to acquire the ratio increase as the occlusion increases, one way ANOVA showed significant difference between each group (n = 9). B) Line of best (LoB) fit for each stent showing the clot weight increase corresponds to an increase in impedance.

The results of blood clot investigation are given in **Figure** **2** and **3**.

### 2.3 Investigating Porcine Vessel

**Figure 4:**
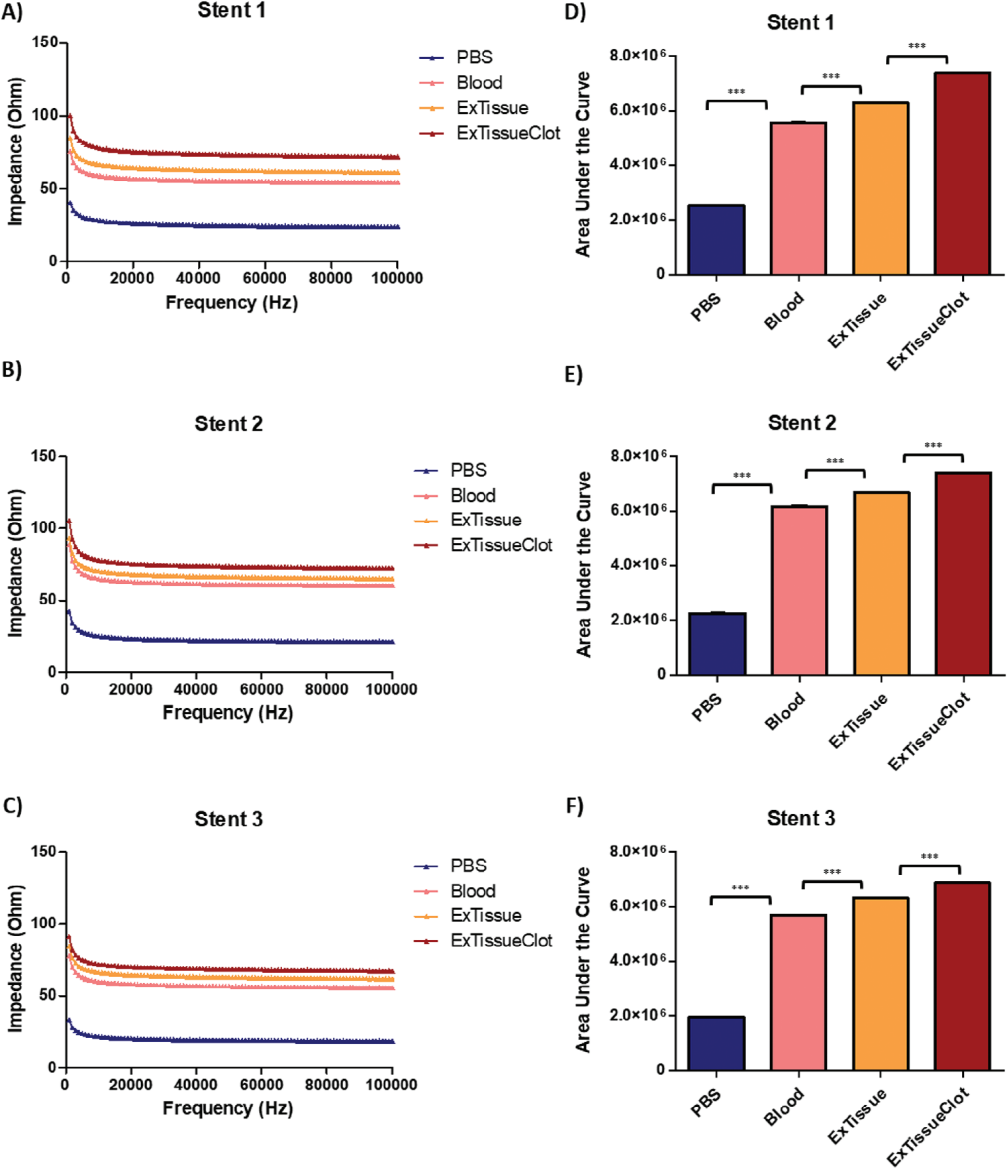
Tissue impedance on whole stent sensors. Frequency sweep and the area under the curve (AUC) results of tissue experiments where (A)–(C) shows the impedance frequency sweep within the ranges of 1 kHz to 100 kHz, and D–F) shows calculated area under the curve. The results confirm that an external tissue around the stent itself contributes to overall impedance of the stent. One way ANOVA showed significant difference (*p* < 0.001) when each group were compared with each other (n = 3).

**Figure 5:**
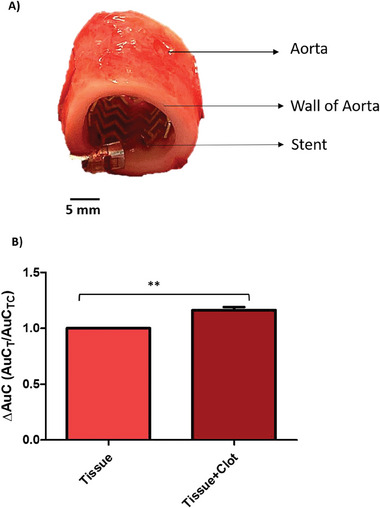
A) showing the stent itself ex vivo before the clot is inserted for testing and B) showing the normalized area under the curve of three different stent experiments. Each stent was tested three times with and without clot while completely surrounded by fresh pig aorta, and result of the frequency sweep was used to calculate the area under the curve. The percentage increase was then calculated by taking the average of each stent experiment and normalizing of the average data with each group`s un‐clotted experiment (as control group). The normalized area under the curve data showed significant difference when two tailed one sample t test was performed for statistical significance to the control group and a 16% +/−3% increase in impedance was detected when the stent was clotted (^**^ *p* = <0.001 *p* = 0.0090, *n* = 9).

The results of porcine vessel investigation are given in **Figures** **4** and **5**.

### 2.4 Adaptation of a PCI Ready Stent

**Figure 6:**
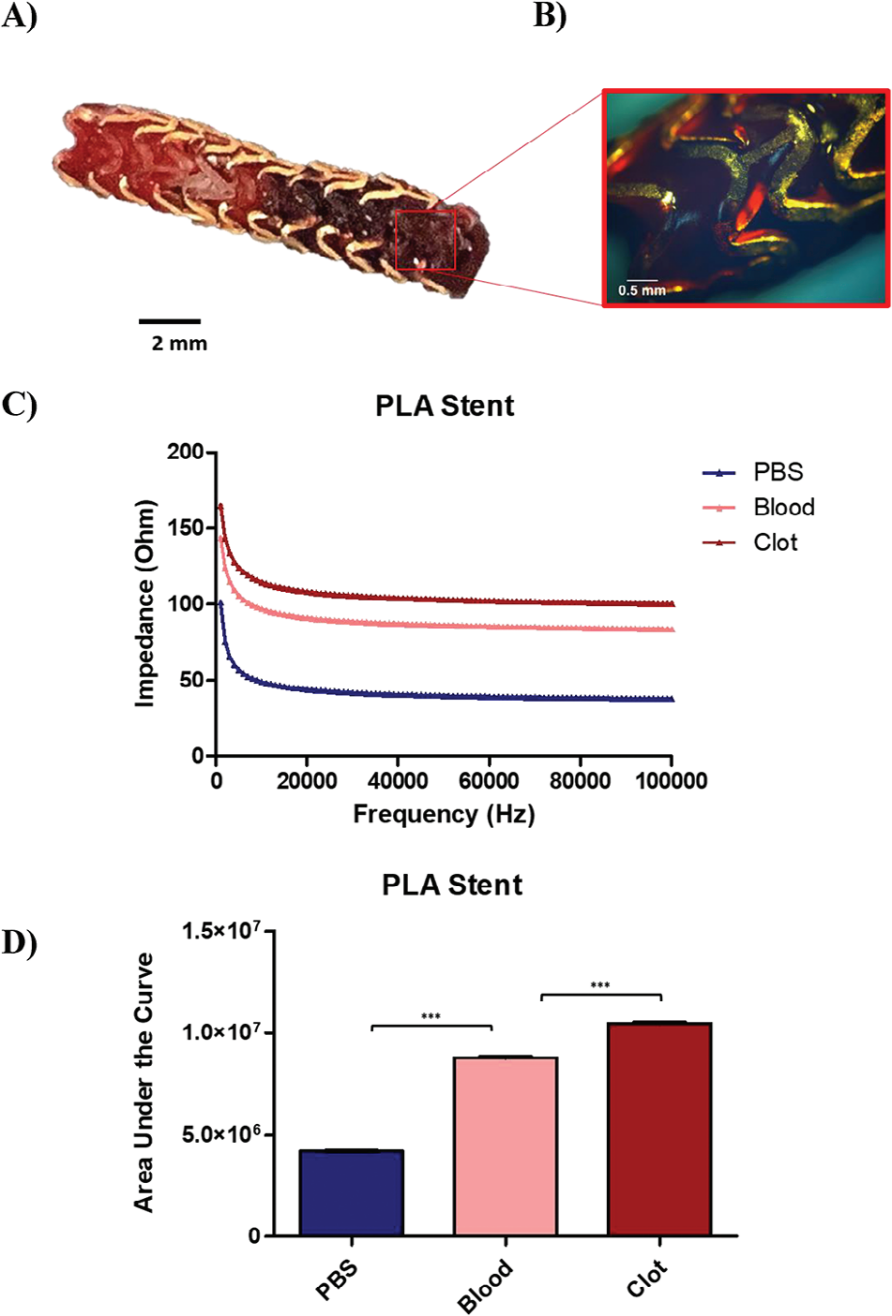
Images and impedance results of a single metallized PLA (Abbott ABSORB GT1) stent. A) and B) shows halfway clotted PLA stent and impedance measurements which demonstrate that the stent was able to detect the occlusion caused by the clot. Impedance frequency sweeps were measured within the ranges of 1 kHz to 100 kHz and used to calculate the area under the curve (D). The calculated area under the curve was then used with one way ANOVA to compare the statistical difference between with and without blood and with and without clot (*p* < 0.0001).

The results of PCI ready stent adaptation are given in **Figure** **6**.

## 3 Discussion

IST and ISR have a profound impact on human health. Developing self‐reporting stents is becoming a reality that can overcome the limitations of existing cardiovascular stent technologies. Previously our group has explored the detection of blockages and cell discrimination by utilizing the changes in electrical impedance.^[^ ^35^, ^36^, ^37^, ^38^ ^]^

In contrast Musick et al. showed a specially engineered piezoelectric microcantilever for a stent that can be used for endothelial cell detection. This novel approach could be essential during recovery from stent deployment to observe the changes in the stenotic region.^[^ ^39^ ^]^ However, due to complex healing mechanisms, the cantilevers detection can be affected by the additional biological materials that are circulating or growing around the stent.^[^ ^31^ ^]^

Pressure sensors have also been widely used for smart stent applications, and significant contributions to the field have come from the Kenichi Takahata group. Their latest adaptation of a smart ureteral stent works with an integrated radiofrequency antenna and micro‐pressure sensor for resonance‐based wireless sensing that is capable of tracking kidney pressure.^[^ ^40^ ^]^ Their cardiovascular approach has a smart stent equipped with microscale sensors and a wireless interface that is capable of continuous monitoring of restenosis formation after stent deployment via a wireless pressure transducer that can track local hemodynamic changes by detecting the different stages of re‐narrowing.^[^ ^21^ ^]^ However, this re‐narrowing needs to reach significant proportions to be detected and doesn't have the ability to reduce the blockage. Another example of this type of pressure sensors is the specifically fabricated stent antenna of Liu et al. that utilizes a pressure sensor for monitoring purposes, as well as the Park et al. approach of creating a sensor integrated with an inductor‐capacitor (LC) circuit that is capable of being wirelessly powered through the changes in pressure which are measured by evaluating capacitance changes.^[^ ^41^, ^42^ ^]^ A different perspective has been presented by Kim et al. arguing that the introduction of sensor packages to the stent through micro‐welding or adhesive bonding could cause complications such as electrical noise, or post deployment sensor detachment from the stent which is a major health risk for the patient. Therefore, they built a self‐rollable stent with an integrated pressure sensor that avoids this and demonstrated its capabilities for both mechanical strength and cellular detection.^[^ ^43^ ^]^ These fascinating smart stents show great promise, but with some drawbacks; the pressure change is small until a significant blockage has appeared on the sensor area location. Additionally, even though they can be used for diagnostic purposes, they cannot, by themselves, be used for therapy to reduce the blockage. In contrast, our work showed that it is possible to track cell growth over time with the ability to induce controlled apoptosis to reduce the number of cells that are growing on the detection area, thus reducing the occlusion.^[^ ^36^, ^37^ ^]^ Moreover, by using the whole stent as a sensor, the detection of occlusion can be observed around a larger area without being limited to sensor integration location. Surface features pre and post metallization modification were assessed using high‐resolution microscopy and future work will assess how these changes impact on hemolysis (Figure S1, Supporting Information).

A different route in smart stents is the use of bioabsorbable stents, which requires the use of biocompatible and bioabsorbable materials for both stent and the sensor itself. For these purposes, Park et al. have created a biodegradable pressure sensor that consists of poly(d‐lactide) (PDLA) integrated to a Polycaprolactone (PCL) based polymer stent that has the ability to be bio‐absorbed.^[^ ^44^ ^]^ However, due to concerns with the ABSORB GT1 system, the clinical use of these kind of bioabsorbable stents were halted.^[^ ^19^ ^]^ Moreover, bioabsorbable smart stents require additional packaging to be bioabsorbable as well. This packaging could be the required telemetry device, or a battery that powers the smart stent. This makes the approach of bioabsorbable smart stent systems to be extremely challenging.

Grafts are another type of vascular implant which are similar to stents which makes them equally suitable for smart development. D'Ambrogio et al. has engineered a novel biocompatible piezocomposite that is suitable for self‐monitoring after graft deployment by monitoring the blood pressure with an extra inductive coil that performs as a resonant sensor, whose resonance frequency responds to the occlusion developments.^[^ ^45^ ^]^ Equally, Natta et al. showed a similar approach with real‐time monitoring of a graft by wrapping an ultra‐thin, biocompatible and flexible smart patch around the graft that detects changes in hemodynamic parameters with high sensitivity of 0.012VPa^−1^ m^−2^.^[^ ^46^ ^]^ The benefit of a graft, with these types of blood pressure or flow sensors is that it can be used without a tethered attachment as the electronics are integrated. However, these devices do not provide any therapeutic options to reduce or control the occlusion. Zhang et al. then showed that a resistance‐type flexible pressure sensor with double conductive layers and crack structure can track blood pressure and capture the pulse movements when it was placed in the radial artery of the human wrist.^[^ ^47^ ^]^ On the other hand, Lu et al. presented a flow sensing system that was capable of detecting blood flow while having a sub‐millimetre scale and multi‐nodal thermal probes. They argued that the existing technology can be disruptive to the normal blood flow and could cause unnecessary tissue damage during the fixation to the vascular or skeletal structures. Therefore, in order to avoid the issue, they have designed biodegradable barbs for the probe to be secured to the tissue around the deployment area, which would allow removal after a certain amount of time.^[^ ^48^ ^]^ Nevertheless, the issue of these devices and sensors being only used for diagnostic purposes remains and they still require a significant blockage or abnormality to occur to provide information.

**Figure 7:**
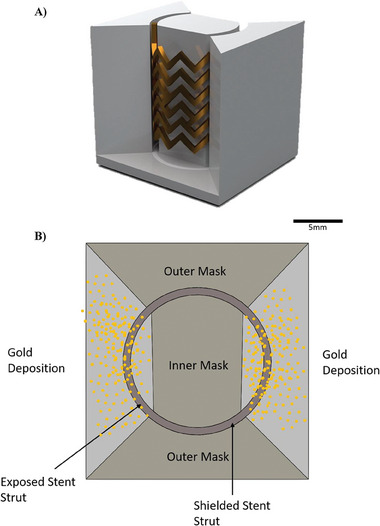
A) Graphical representation and B) schematic top view representation of shadow masking; the Stent is placed within 3D printed masks which selectively shields areas of the stent from being metallized. Both the outer and inner parts of the mask block the metal deposition to the stent struts while the exposed areas are coated with gold. This masking effect then converts exposed struts of the stent into electrodes.

**Figure 8:**
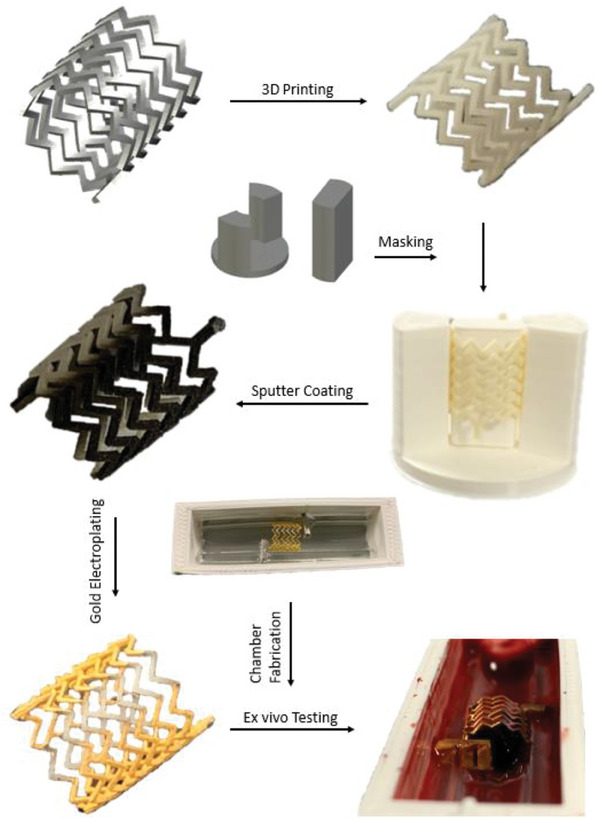
The process of converting the whole stent into a sensor starts with 3D model creation; the model was 3D printed using biocompatible resins and specific areas of the 3D print were shielded to establish a seeding layer with sputter coating. This process converted the inert stent to a set of electrodes across which impedance can be measured creating a whole stent sensor. Additionally, specifically tailored testing chambers that are highly adaptable and convertible to be used with any stent type were fabricated to accommodate the stent during experiments.

A fast way of prototyping a smart stent is with 3D printing technology. This 3D printing must be completed using a biocompatible material in order to keep the stent from being harmful in vivo. We have shown how shadow masking can be used to partially metallize the stent, creating two conductive elements (**Figure** **7**). This novel approach to the creation of a sensor can be applied to any non‐conductive material, which makes it applicable to current drug eluting stents to convert an already approved stent into a sensor for diagnostic purposes. Additionally, the unique approach of creating a testing system means the work can be applied to different stents with different geometric dimensions and structural differences. The testing chambers are easily modifiable and highly interchangeable due to the utilization of 3D printing and molding, which allows *ex vivo* testing of different systems with small changes **Figure** **8**.

Simulations were necessary to understand how the electric fields form within the stent and to understand what effect the stent material had with the biological material. For this purpose, a COMSOL simulation was created to observe the cross section of a stent in blood with and without a blockage. The COMSOL simulations (Figure 1) showed that distortion to the electric fields happens once an area within the stent was changed from blood to blood clot. This caused a change in the electrical impedance measured with the less conductive blockage producing an impedance increase.

Our experiments, that were conducted using heparinized porcine blood and blood clot, closely mirrored the results from the COMSOL simulations. However, the area under the curve did show some expected variance between Stent 1, 2 and 3. This was caused by unique differences in the stents during the metallization process as each stent had a custom testing chamber fabricated to be used in their respective set of experiments. Nevertheless, each stent showed a significant difference to its control. Furthermore, the significance was maintained even when stent groups were combined to be analyzed across biological repeats.

The ex vivo experiments with porcine aorta showed that the stent's impedance during measurements was statistically affected by the presence of the tissue cuffed around the stent (Figure 4). Importantly the impedance was not totally dominated by the surrounding tissue, as the stent was still capable of detecting occlusion caused by the clot formation with a 16% +/−3% increase in impedance. The area under the curve of the clotted stent showed significant difference (p = 0.0090) when analyzed using two tailed one sample t test (Figure 5). This corroborates that after deployment the stent will be able to detect occlusion and its formation over time within a vessel.

Our previous experiments were conducted using a special, biocompatible 3D printed stent (10 mm x 10 mm). In order to explore if the methodology can be used with any stent, a commercially available Abbott ABSORB GT1 (12 mm x 3 mm) stent was chosen due to its non‐conductive base material. The stent was selectively metallized to be used in our experiments. Modifications to testing chambers were applied and a metallized Abbott ABSORB GT1 stent was tested with and without blood clot occlusion, which showed significant differences between each group (Figure 6). This experiment showed that any suitable non‐conductive stent can be converted to a “smart” stent with selective metallization methodology, although this will have implications on the biodegradability of the stents designed to be naturally absorbed.

These results indicate that these types of modification to existing stents can detect and differentiate different sized blood clots and detect these clots within the aorta. Therefore, it is expected any acute blood clot formation would be detected by the stent either immediately post‐deployment, or in later chronic stages after deployment. We have seen significant variation in impedances between devices and expect every stent will have their unique impedance when they are prepared, and when they are deployed (or tested *ex vivo*) the immediate impedance measurement can be considered the baseline or starting point. Hence, the earliest signs of any impedance increase after the deployment will give insight into the cellular development at the location, and this effect should be observed in each individual stent that is used/deployed with patients. Additionally, due to the whole stent acting as a sensor, the detection will be observed across the entire area of the stent, rather than being limited to a specific region. Moreover, the stent can be separated into more than 2 electroplated segments to improve localized detection by converting the stent to have more than 2 sets of electrodes. Previously our group showed that the same technology can be used with IDE (interdigitated electrode) sensors. These studies have shown that electrical impedance can give real time monitoring of the state of a device with the frequency of the measurements being determined by the measurement electronics. The use of the whole stent as a sensor element but also integrated with multiple IDEs creates a hybrid smart stent system. This hybrid system has the advantage of intrastent clot detection for early diagnosis but with the advantage of therapeutic cell killing.^[^ ^36^ ^]^

Drug eluting stents are constructed from a metal stent coated with a polymer that contains the drug. For the impedance sensing technique, we have developed, to work the surface of the stent must have some conductive and some non‐conductive areas and if this is achieved by selectively coating a drug eluting stent with gold electrodes the gold film would interfere with the drug delivery. It could be envisaged that a stent featuring both technologies could be created that has some areas with active sensors and other areas coated with a drug eluting coating.

It should be noted that the experiments conducted in this work were performed with stationary blood and it is known that ionic fluids do show a small increase in electrical impedance when flowing however the increase is small at the frequencies we have studied and disappears at frequencies over 100 kHz, where we still show an increase in impedance due to clot, which shows this sensing mechanism would function in flowing blood.^[^ ^49^ ^]^

In our study, we have discovered that preparing the stents for experiments requires significant time. This is mainly due to the laborious process that needs to be conducted to create an electroplated whole stent sensor. Moreover, the stent`s frigidity has caused complications during experiments and the different electrical properties of the stents and testing chambers have introduced extra variation to the results. Nevertheless, the 3D printing can be adapted very rapidly to overcome functional issues with the experimental setups. An additional limitation we have found during our study was the relatively large size of the stent due to limitation of 3D printing. However, we have shown that a previously commercially available stent can also be used with the same methodology. Even though this methodology requires the stent to be non‐conductive, we believe that any stent can be used in this whole stent sensing approach by coating the stent with a Parylene C coating, thus converting the naturally conductive stent to a non‐conductive surface.

In order for the stent to be deployed and relay data to outside the body, our group has developed an Implantable Impedance Telemetry (IIT) device that can be used with our sensors. We eventually propose to advance the developments on this telemetry device to be an ASIC system (Application Specific Integrated Circuit). In addition, patient‐specific 3D printing could be used to tailor the stent geometry to the patient. There is also precedent of implantable telemetry units being connected distant from the implant by threading the connection lead through a vein, such as found in Stentrode Technology.^[^ ^50^ ^]^

We believe that the novel approach we have shown here that converts an inert medical device into a biosensor through selective metallization can potentially be applied to a variety of other medical devices.

## 4 Conclusion

Stent thrombosis is a major issue with PCI and stents and could occur after stent deployment or even in later stages. In this paper we have shown that a stent can be selectively metallized to be used for clot detection. For this purpose, a COMSOL simulation was created for a stent to show if it is possible for a material to have an effect on the electrical impedance measured across sensors on the stent, which showed that an addition of a material (clot) that has different electrical properties than the surrounding media (blood) can be measured by the whole stent sensor. The experiments showed that these stents can detect different percentages of clot and can detect an occlusion while surrounded by porcine aorta. Additionally, we have shown that this methodology can be used with a suitable commercially available stent, which converts the inert stent into a “smart” stent that is capable of clot detection through changes in impedance around the stent itself. This promises great potential for use with vascular implants and indeed other medical implants that could benefit from the measurement of the build‐up of biological material in their vicinity.

## 5 Experimental Section

### 3D Stent Preparation

SolidWorks (Massachusetts, USA) has been used to prepare the 3D model for printing. Stent designs were fabricated using a biocompatible ink MED‐WHT‐10, ISO 10993–5 and ISO 1993‐10, and printed using a stereolithographic 3D printer (3D Systems, California, USA, Figure 4). A scaffold and a mask to allow selective metallization through shadow masking was prepared using SolidWorks (Massachusetts, USA) and 3D printed using FDM 3D printer in PLA.

These scaffolds were then used during the metallization process to shield specific parts of the stent to make it partially coated. An Agar Sputter Coater (Agar Scientific, Stansted, UK) was used in cycle setting where, the complete sequence of flush, coat and vent is automated. The sputter coater coated the stent with 108 nm gold:palladium thickness (80:20 ratio). After sputter coating, a Fluke 3000 FC Multimeter was used to test the conductivity of the coated stents. Sputter coating of the stents prepared the required “seed” layer for electroplating. Gold, non‐cyanide containing electroplating solution (ECF 60 ready‐to‐use solution, Gold Solutions Plating, UK) was then warmed to 50 °C and agitated using an electronic mixer for ≈1 h inside of a beaker within a water bath. Once the solution was at a sufficient temperature, the stent was then connected to a power source and submerged in the electroplating solution and plated for 10 min under 2 V 20 mA conditions. After both conductive parts of the stent were electroplated with gold, the conductivity of the electroplated stent was checked via Fluke 3000 FC Multimeter for continuity across the whole stent. The selective electroplating was specifically targeted to the bottom and top parts of the stent to improve the stability of the experiments by manipulating the electric fields to cover the intraluminal stent space.

### Testing Chamber Preparation

Preparation of the testing chamber required a two‐stage process; 3D printing of the inner chamber and sterile mounting of the chamber with PDMS (Polydimethylsiloxane, SYLGARD 184, 1.1KG, Farnell, Illinois, USA) casting. The former was achieved by using a FDM printer and was printed using PLA as the base material. The print was then bonded to glass slides using ultraviolet curable glue (Loctite AA 350 UV, Henkel Limited, Germany) on a UV stage for ≈10 min until the glue was solid. For the latter, uncured PDMS was mixed with its curing agent at 10:1 ratio and degasified for 30 min to remove any extra air bubbles from the uncured PDMS. This was then followed by placing a 3D printed PLA sacrificial core with connectors to the testing chamber which after removal would create a curved shape for the stent to fit into. PDMS was poured and baked on a hot plate for ≈1 h at 75 °C to achieve the desired shape for the testing chamber. The sacrificial core was then removed once the baking was finished. In order to achieve a connection from the testing chamber to the LCR meter, multi core wires were connected to the connectors through inlets on the testing chamber. The connection was then further enhanced via soldering and stabilized with UV curable glue.

### COMSOL Simulations

Simulations were created using COMSOL Multiphysics v 5.5 (Stockholm, Sweden) to understand how the electric fields were affected by the presence of a biological material. This was achieved by building a cross section of a stent in SOLIDWORKS and importing the drawing into COMSOL where multiple materials and their properties were assigned to the locations of the stent using electrical properties of blood and clot.^[^ ^51^, ^52^ ^]^ Once the model was prepared, the fields were modelled from 1 kHz to 10 MHz frequency range. In COMSOL the AC/DC package was used to solve Maxwell's equations across the set geometry whilst using the quasi‐static approximation.

### Experimental Setup

Fresh porcine blood clot and aorta were obtained from Glasgow Veterinary School under license from the NHSGGC Biorepository and Pathology Services (REC 10/S0704/60) after approval from the West of Scotland Research Ethics Service. Stents and testing chambers were cleaned with 70% ethanol and rinsed 3 times with de‐ionized water afterwards to remove any residual ethanol. After the cleaning process, stents were then placed in situ within each testing chambers and electrical continuity was confirmed.

A secondary control that tested the electrical connection security between the testing chamber and stent was performed by filling the chamber with 8 milliliter (ml) PBS (Phosphate Buffered Saline, ThermoFisher, USA), which was used in the initial impedance measurement of the stent. Once the connection had been proven to be secure, 8 ml heparinized blood was then used to fill the chamber and impedance data of heparinized blood was taken as the control group. The medium was changed during each measurement, and a total of 9 replicate measurements were taken. Following this, a small piece of clot was separated from the main clotted blood, its weight measured and then placed carefully to occupy ≈25% of the intraluminal stent space to form and mimic an occlusion. The clot was cut specifically in a longitudinal size to mechanically trap itself within the intraluminal stent space. With the clot in situ, the chamber once again was filled with 8 ml heparinized blood and impedance measurements were repeated. This process was then followed for the next experimental group at increasing occlusions of ≈50%, ≈75% and ≈100%, (n = 3). A similar protocol for the PLA stent (ABSORB GT1 Abbott) was performed, using PBS followed by heparinized porcine blood. This stent being a clinical grade stent it was not possible to fractionate the size of the blood clot with any accuracy so was then occluded to ≈50% for comparison.

To model extravascular field effects, a whole vessel cuff (porcine aorta) was wrapped around the stent in the presence and absence of whole blood and clots were tested with (n = 3).

### Data Collection and Data Analysis

Impedance data were captured using a Hioki IM3536 LCR meter (Hioki Corporation, Japan) with frequency range set up from 1 kHz to 100 kHz. Data analysis and the calculation of area under the curve was conducted via GraphPad Prism (San Diego, USA). All data presented are representative of replicate data sample`s area under the curve with as mean ± standard deviation (SD). One way analysis of variance (ANOVA) and two tailed t‐test was used to calculate the significant difference between multiple groups. Statistical significance was displayed as *p*< 0.05 (one star), *p*< 0.01 (two stars), or *p*< 0.001 (three stars).

## Conflict of Interest

The authors declare no conflict of interest.

## Supporting information

Supporting Information

## Data Availability

The data that support the findings of this study are available from the corresponding author upon reasonable request.
