# Supplementary material for: Detection of Blood Clots Using a Whole Stent as an Active Implantable Biosensor
Source: Adv Sci (Weinh). 2024 Feb 11;11(21):2304748. doi: 10.1002/advs.202304748 (PMC11151072; doi:10.1002/advs.202304748)
Supplement: Supplementary file 1 — Supporting Information [file ADVS-11-2304748-s001.pdf]

ADVANCED SCIENCE
Open Access

Supporting Information

for *Adv. Sci.*, DOI 10.1002/advs.202304748

Detection of Blood Clots Using a Whole Stent as an Active Implantable Biosensor

Mahmut Talha Kirimi, Daniel Hoare, Michael Holsgrove, Jakup Czyzewski, Nosrat Mirzai, John R. Mercer and Steve L. Neale**

Supporting Information

A)

B)

C)

D)

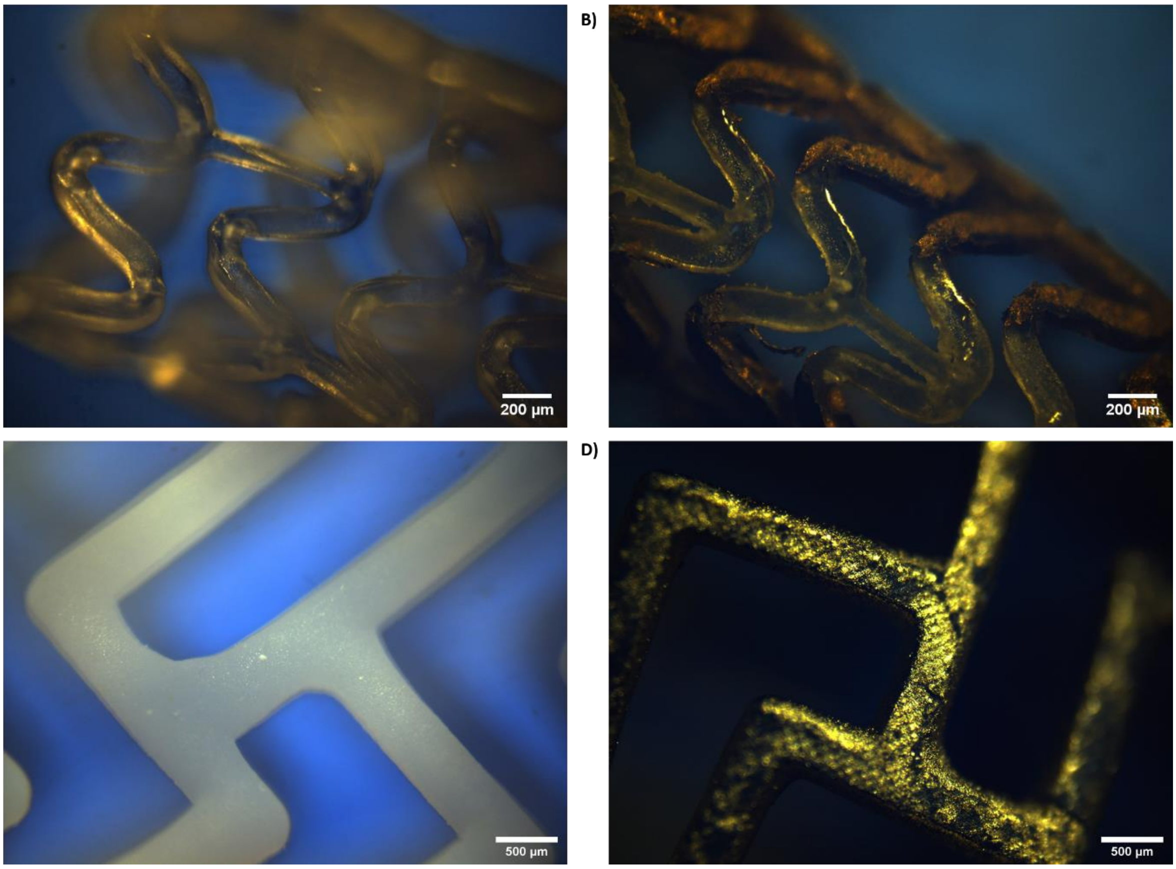

Figure S1. High resolution microscope images of the PLA stent (Abbott ABSORB GT1) and the 3D Printed stent where A) and C) shows pre-metallization and B) and D) shows post metallization surfaces of the stents.
